# Is there evidence of sustained human-mosquito-human transmission of the zoonotic malaria *Plasmodium knowlesi*? A systematic literature review

**DOI:** 10.1186/s12936-022-04110-z

**Published:** 2022-03-17

**Authors:** Pablo Ruiz Cuenca, Stephanie Key, Kim A. Lindblade, Indra Vythilingam, Chris Drakeley, Kimberly Fornace

**Affiliations:** 1 Centre for Health Informatics, Computing, and Statistics (CHICAS), Lancaster University Medical School, Lancaster, UK; 2 Faculty of Infectious and Tropical Diseases, London School of Hygiene and Tropical Medicine, London, UK; 3 Institute of Biodiversity, Animal Health and Comparative Medicine, University of Glasgow, Glasgow, UK; 4 Global Malaria Programme, World Health Organization, Geneva, Switzerland; 5 Department of Parasitology, Faculty of Medicine, Universiti Malaya, 50603 Kuala Lumpur, Malaysia

**Keywords:** Malaria, *Plasmodium knowlesi*, Non-zoonotic transmission, Zoonoses, Macaques, Simian malaria, Systematic literature review, Disease emergence

## Abstract

**Background:**

The zoonotic malaria parasite *Plasmodium knowlesi* has emerged across Southeast Asia and is now the main cause of malaria in humans in Malaysia. A critical priority for *P. knowlesi* surveillance and control is understanding whether transmission is entirely zoonotic or is also occurring through human-mosquito-human transmission.

**Methods:**

A systematic literature review was performed to evaluate existing evidence which refutes or supports the occurrence of sustained human-mosquito-human transmission of *P. knowlesi*. Possible evidence categories and study types which would support or refute non-zoonotic transmission were identified and ranked. A literature search was conducted on Medline, EMBASE and Web of Science using a broad search strategy to identify any possible published literature. Results were synthesized using the Synthesis Without Meta-analysis (SWiM) framework, using vote counting to combine the evidence within specific categories.

**Results:**

Of an initial 7,299 studies screened, 131 studies were included within this review: 87 studies of *P. knowlesi* prevalence in humans, 14 studies in non-human primates, 13 studies in mosquitoes, and 29 studies with direct evidence refuting or supporting non-zoonotic transmission. Overall, the evidence showed that human-mosquito-human transmission is biologically possible, but there is limited evidence of widespread occurrence in endemic areas. Specific areas of research were identified that require further attention, notably quantitative analyses of potential transmission dynamics, epidemiological and entomological surveys, and ecological studies into the sylvatic cycle of the disease.

**Conclusion:**

There are key questions about *P. knowlesi* that remain within the areas of research that require more attention. These questions have significant implications for malaria elimination and eradication programs. This paper considers limited but varied research and provides a methodological framework for assessing the likelihood of different transmission patterns for emerging zoonotic diseases.

**Supplementary Information:**

The online version contains supplementary material available at 10.1186/s12936-022-04110-z.

## Background

Zoonotic malaria caused by the parasite *Plasmodium knowlesi* has increasingly become a public health concern across Southeast Asia [1]. Carried by long and pig-tailed macaques (*Macaca fascicularis* and *Macaca nemestrina*) and transmitted by the *Anopheles* Leucosphyrus Group of mosquitoes, the geographical range of *P. knowlesi* is limited to areas where both the primate hosts and vectors are present [2, 3]. Although *P. knowlesi* was first identified and isolated in the 1930s, naturally acquired human infections were believed to be rare until a large cluster of human infections was identified in 2004 in Sarawak, Malaysian Borneo [4]. While human cases of *P. knowlesi* were initially primarily reported in individuals living or travelling through forest and forest areas, increasing evidence suggests wider distribution of infections in individuals without occupational exposures [5–7]. Since then, *P. knowlesi* infections have been reported across Southeast Asia, most notably within Malaysia [8–10]. Malaysia has now eliminated all indigenous non-zoonotic malaria transmission, but *P. knowlesi* incidence continues to rise, threatening to undermine Malaysia’s malaria elimination goals [9].

Although the majority of *P. knowlesi* transmission is believed to be zoonotic spillover, one of the key outstanding questions is whether *P. knowlesi* is transmitted from human infections to other humans. Zoonotic emerging infections may be classified into 5 stages based on epidemiological dynamics in the incidental host [11]. Stages 1 and 2 represent pathogens which are found in animals and have either not been found to naturally infect humans or have not been found to cause secondary human infections, only infecting humans through direct zoonotic spillover. Stage 3 pathogens, such as SARS-CoV-1, MERS-CoV and Monkeypox, are weakly transmissible between humans and cause stuttering chains of transmission that ultimately die out or are controlled. Stage 4 represents those pathogens that have a natural cycle of infecting humans from the primary animal host and can produce long sequences of secondary human cases. These are divided further into subgroups (4a, 4b, 4c) according to the importance of transmission within the reservoir or incidental host [11]. To illustrate, yellow fever is considered a Stage 4a pathogen because its sylvatic transmission cycle is more important to maintaining pathogen transmission than non-zoonotic spread, whereas influenza A and cholera are considered Stage 4c pathogens because their transmission is primarily between humans [12]. These zoonotic transmission cycles can present challenges for control and elimination. For example, despite an effective vaccine for yellow fever, zoonotic transmission dynamics have proved to be a major barrier to elimination and are the reason yellow fever eradication was determined to be infeasible [13].

Similarly, ascertaining the status of *P. knowlesi* as a zoonosis or human pathogen is critical to determining the overall feasibility of malaria eradication and shorter-term goals of malaria elimination in Southeast Asia. In 2017, a World Health Organization (WHO) Expert Review Group (ERG) examined available evidence to determine whether sustained human-mosquito-human transmission of *P. knowlesi* was occurring. As there was limited evidence of sustained non-zoonotic transmission following an initial spillover from wildlife, the ERG classified *P. knowlesi* as a primarily zoonotic infection (Stage 2), but highlighted the need to investigate the potential for non-zoonotic transmission [14].

Human infection with *P. knowlesi* is already conclusively documented. However, there remain two barriers to sustained human-mosquito-human transmission: (a) the parasite’s ability to reproduce asexually within the human host and produce sufficient, viable gametocytes capable of generating infection in the mosquito vector, and (b) the limitation of sexual reproduction of *P. knowlesi* to mosquitoes of the Leucosphyrus Group, which restricts transmission to zones where both humans and these vectors are abundant. If human-mosquito-human transmission is limited by the life cycle of the parasites in humans, then *P. knowlesi* can be considered a Stage 2 zoonosis and not a human parasite. However, if sustained non-zoonotic transmission is limited only by the range and density of the vector, then *P. knowlesi* may be considered an emerging human malaria parasite with the potential for increasing transmission if the mosquito’s behaviour or ecological niche changes or if the parasite adapts to become able to complete its extrinsic cycle in other malaria vectors.

Four parameters determine *P. knowlesi* transmission: interactions between human host and parasite (e.g. parasite virulence, human-to-mosquito infectiousness, parasite binding to human red blood cells), interactions between human host and vectors (e.g. human host biting rates, biting preferences, ratios of mosquitoes to human hosts), interactions between vectors and parasite (e.g. mosquito lifespan, ability of the parasite to reproduce in mosquitoes, duration of parasite development) and vector and wildlife host ecology. Changes in any of these parameters can influence the zoonotic potential and/or the basic reproduction number (R_0_) of *P. knowlesi* between humans. To evaluate evidence of sustained human-mosquito-human transmission, the ERG identified key categories of evidence influencing these parameters which would either support or refute non-zoonotic transmission [14]. These include epidemiological, ecological and laboratory studies characterizing these transmission parameters, as well as broader evidence on the distribution of *P. knowlesi* infections in humans, simian hosts and mosquito vectors. Additionally, collation of these key parameters can be used to refine modelling approaches in order to evaluate the contributions of zoonotic and non-zoonotic transmission pathways from routinely collected surveillance data [15, 16].

## Methods

### Evidence of human-mosquito-human transmission

The primary objective of this systematic literature review is to assess evidence of sustained non-zoonotic transmission. First, categories of evidence which could support or refute the occurrence of sustained human-mosquito-human transmission were identified. These consist of evidence categories identified by the ERG and refined further in consultation with the WHO, detailed in Table 1 [14]. The strengths of the evidence were determined based on consultations with the WHO and key stakeholders, and expert opinion.

**Table 1:**
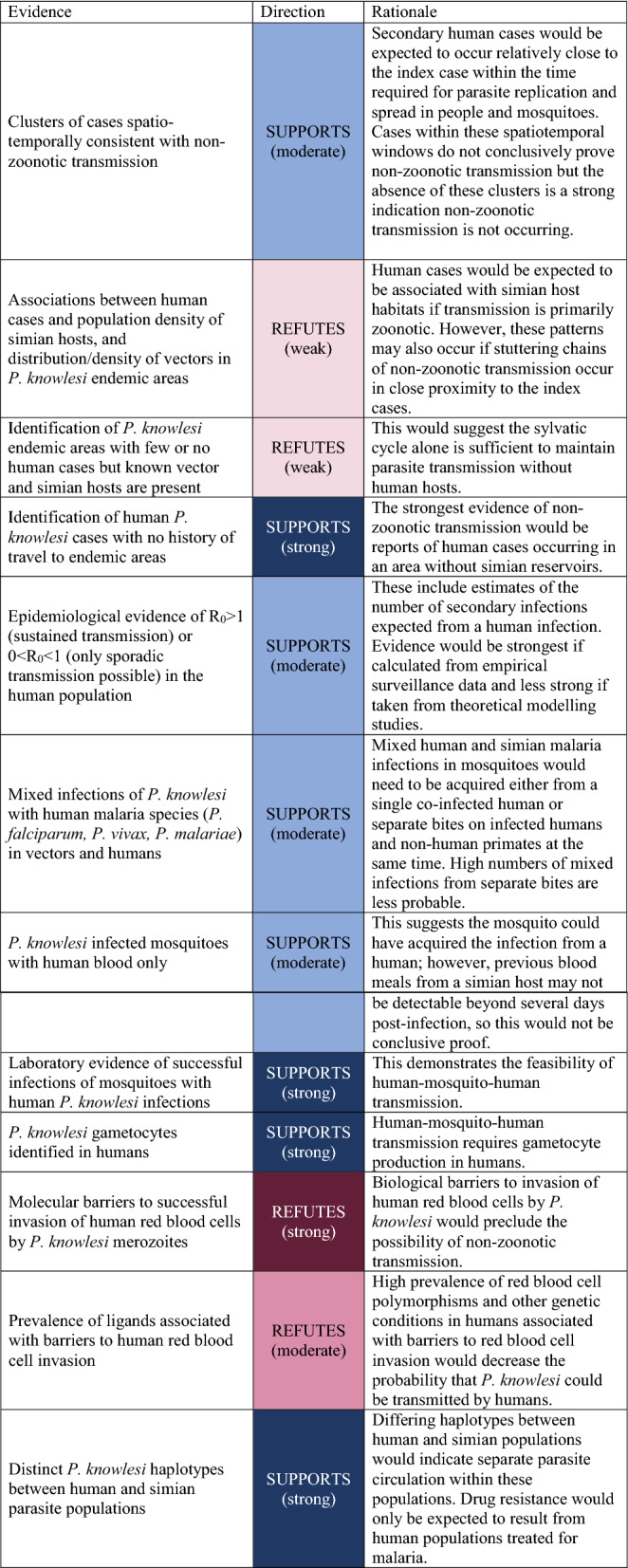
Evidence identified by the previous ERG, showing potential data sources and how each type of evidence would support or refute non-zoonotic transmission

These transmission mechanisms are highly dependent on the environment and distribution of human infections, simian hosts and mosquito vectors. Therefore, additional evidence categories relevant to transmission but not directly supporting or refuting human-mosquito-human transmission were also identified. These included spatial and temporal data on the distribution of cases, distribution, species and infections in mosquito vectors, and abundance and prevalence in simian hosts.

The full protocol for this systematic literature review is included in Additional file 1: Appendix 1.

### Eligibility criteria

The CoCoPop (Condition, Context, Population) framework used for incidence and prevalence studies was adapted to help develop the main research focus of the systematic literature review [17]. This framework was selected because it is suitable for using contextual data, such as presence and abundance of vectors and hosts, in the analysis of incidence and prevalence data. These eligibility criteria were as follows:

#### Types of study

All types of study were included, except for literature reviews with no primary data collection or analysis. Other studies were included to identify information from the broad range of evidence highlighted by the ERG [14]. Conference proceedings and abstracts without full methodologies and verifiable data were excluded.

#### Language

The literature search was completed in English, but all literature sources in Bahasa Malay/Indonesian, French, Portuguese and Spanish were also considered. Literature sources in other languages were excluded.

#### Condition

The condition was defined as zoonotic malaria, focusing on *P. knowlesi*. Zoonotic malaria caused by other *Plasmodium* species in Asia (e.g. *Plasmodium cynomolgi*) was also included because research on other simian *Plasmodium* species could be suggestive of sustained human transmission. Zoonotic malaria species not endemic to Asia (e.g. *Plasmodium simium* in South America) was excluded.

#### Context

Due to the complexity of studying transmission dynamics and broad range of evidence needed to support or refute sustained non-zoonotic transmission, studies carried out in controlled laboratory conditions were included along with field studies and mathematical modelling studies. Locally transmitted *P. knowlesi* has only been reported in Southeast and South Asia, so studies for which the context was applicable to this region were focused on. However, in line with the condition criteria above, studies from other locations were also considered if relevant information was included (e.g. case reports and experimental studies).

#### Population

No exclusion criteria were applied to the populations used in this review.

### Information sources and search strategy

Due to the broad range of evidence, a wide search strategy was employed. This search strategy was reviewed by an Information Scientist from the Library at the London School of Hygiene and Tropical Medicine using the Peer Review of Electronic Search Strategies (PRESS) standards and guidelines [18]. Additionally, the protocol was peer-reviewed by *P. knowlesi* experts in genetics, immunology, molecular biology, entomology, primatology and epidemiology.

The following databases were used to search for published research: Medline, EMBASE, and Web of Science.

The search strategy used is outlined below:

1. *Plasmodium knowlesi*/
2. plasmodium knowles*.mp.
3. 1 or 2.
4. Zoonoses/
5. haplorhini/ or catarrhini/ or cercopithecidae/ or cercopithecinae/ or exp macaca/
6. (monkey* or simian* or zoono* or macaca or macaque*).mp.
7. 4 or 5 or 6.
8. (malaria* or plasmodium).mp.
9. Malaria/
10. 8 or 9.
11. 7 and 10.
12. 3 or 11.

### Systematic literature review

The literature search was carried out on 4th January 2021, and all results were imported into EndNote (Clarivate Analytics, London, UK). An extensive deduplication process was used to remove all duplicate references. The remaining references were transferred into Rayyan **(**https://www.rayyan.ai/**)** [19], an online tool designed to facilitate title and abstract screening. The criteria above were used to include and exclude papers based on potential relevance to the categories of evidence identified. An initial reviewer screened all reference abstracts, and a second reviewer screened all abstracts excluded by the first reviewer.

The synthesis without meta-analysis (SWiM) framework was used because of the wide range of evidence assessed [20]. This framework applied vote counting to synthesize evidence using a standardized form to categorize the evidence from each reference (Appendices 1–2). Based on the categories described in Table 1, evidence suggestive of sustained human transmission was considered a positive vote (up), and evidence refuting non-zoonotic transmission was considered a negative vote (down). The study type was recorded along with and the diagnostic methods used, if relevant. References were classified into the following categories of evidence: human infections, simian infections and distributions, vector infections and distributions, parasite genetics, and transmission dynamics, including invasion pathways.

For all full references included, a vote was assigned for whether the evidence supported, refuted, or was neutral (did not support or refute) on non-zoonotic *P. knowlesi* transmission. The main findings of the article were also summarized. These data were presented in narrative and tabular form using vote counting. The synthesis was discussed amongst reviewers and subject experts before final results were agreed.

**Fig. 1:**
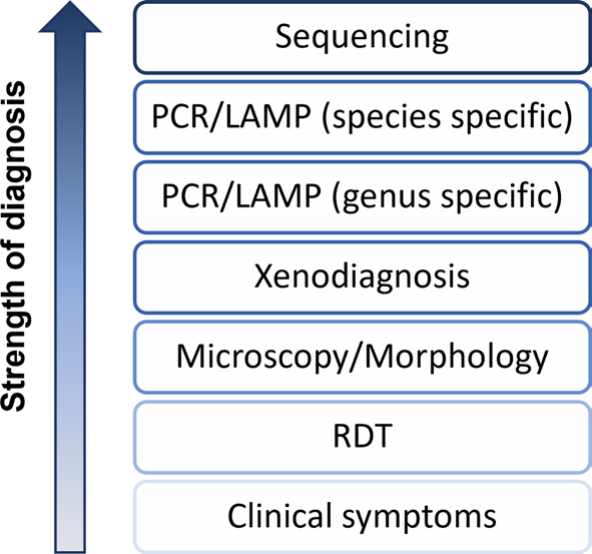
Qualitative assessment of diagnostic methods to identify *P. knowlesi* infection in humans. PCR/LAMP are separated into two categories due to the reported cross-reactivity between *P. vivax* primers and *P. knowlesi* samples

Given the comprehensive nature of the evidence being searched, no risk of biases or meta-biases from the studies was assessed. Instead, the reliability of each study was assessed qualitatively and graded as reliable or unreliable. For studies reporting *P. knowlesi* infections, this reflected the accuracy of the diagnostic methods (Fig. 1). Additionally, consideration was given to other methods which were applicable to each type of study and how these were reported alongside results in the final manuscript. The assessed methods included epidemiological study design, sample selection, genetic extraction and amplification, modelling techniques and replicability [21, 22].

**Fig. 2:**
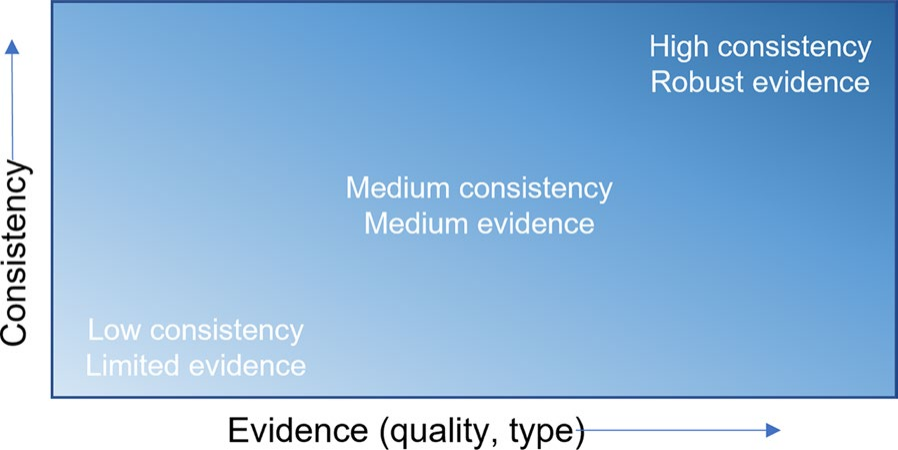
Evidence and consistency statements and their relationship to confidence. Confidence increases towards the top-right corner as suggested by the increasing strength of shading

In the process of synthesizing the available evidence, key findings for each of the categories were summarized. The evidence was also qualitatively classified, following the same guidance provided by the Intergovernmental Panel on Climate Change (IPCC) (Fig. 2, [23]).

## Results

### Literature assessed

**Fig. 3:**
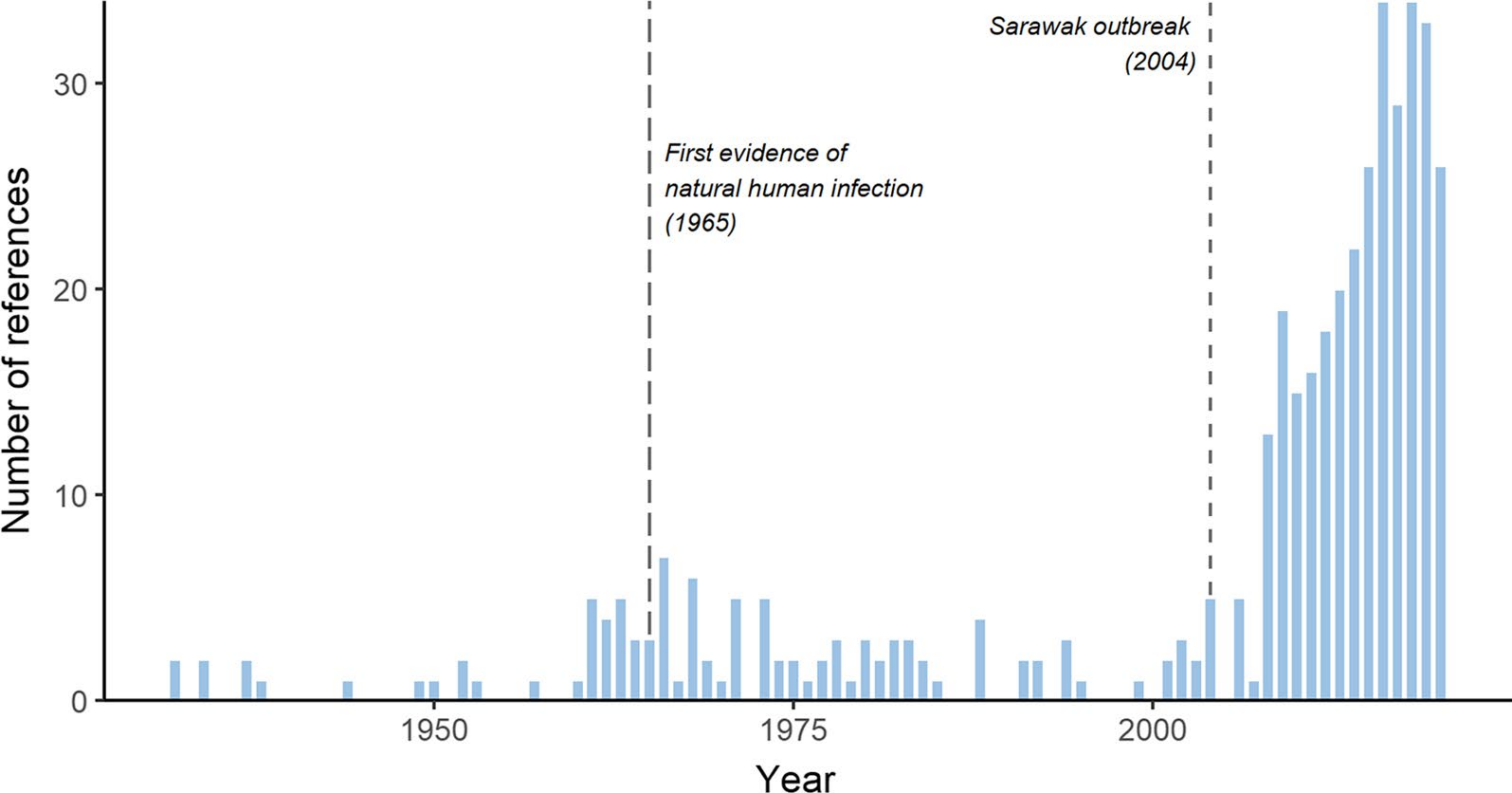
Number of references retrieved by year. Vertical dotted lines represent 2 key points in time: the first report of natural human infection with *P. knowlesi* and the identification of a *P. knowlesi* outbreak in Sarawak, Malaysian Borneo

**Fig. 4:**
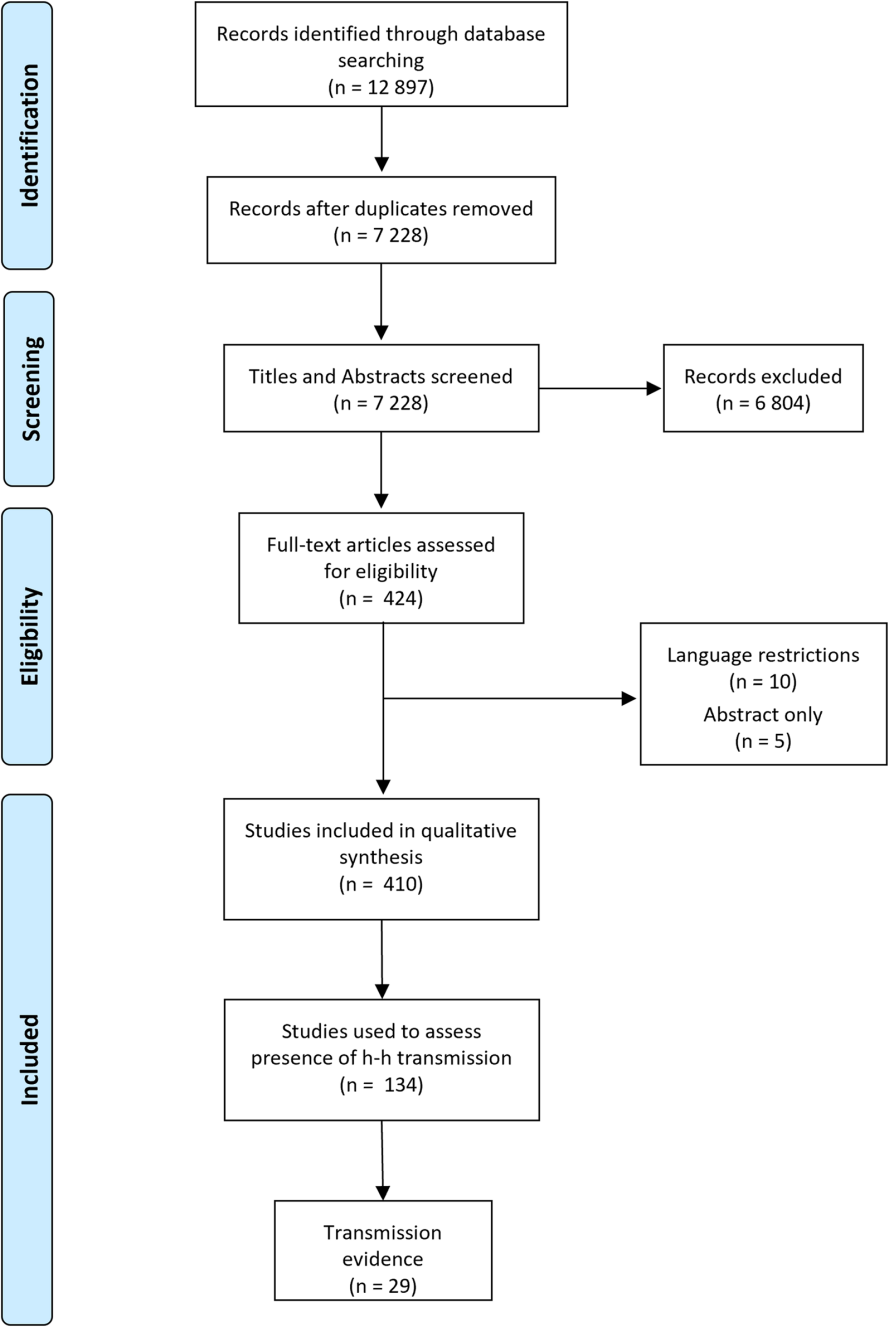
PRISMA flow chart, indicating number of references included and excluded at various stages

The initial search strategies used in the selected research databases yielded a total of 12,897 records. After an extensive de-duplication process, 7,229 records remained for title and abstract screening. Of these, 6,813 were excluded using the exclusion criteria detailed in the methods section. The remaining 424 references were used to source full texts, and these were assessed for eligibility. From the final screening of all 424 full papers, 409 were included in the final literature review (Figs. 3, 4). Of these, only 29 were deemed to directly support or refute human-mosquito-human transmission and included in the graphical synthesis. A total of 87 references were identified which described infections in humans, 13 describing infections in mosquitoes and 14 describing infections in simian hosts (Additional file 2: Appendix 2, Additional file 3: Appendix 3, Additional file 4: Appendix 4). These references provide critical information about the distributions of infections and presence of mixed infections, but they do not directly address the question of human-mosquito-human transmission.

### Evidence of sustained human-mosquito-human transmission

After extensive deduplication and filtering, a total of 131 studies were included in the evidence assessment, 29 of which were deemed to directly contribute evidence which supported or refuted non-zoonotic transmission (Table 2). 18 studies supported sustained non-zoonotic transmission across the various evidence categories. One showed spatio-temporal clusters indicating possible human-mosquito-human transmission, one provided evidence from mathematical modelling studies, four showed mixed plasmodium infections in known vectors, one showed successful experimental human-mosquito-human transmission, seven identified invasion pathways into human red blood cells, and four described possible distinct haplotypes between human and simian infections.

**Table 2:**
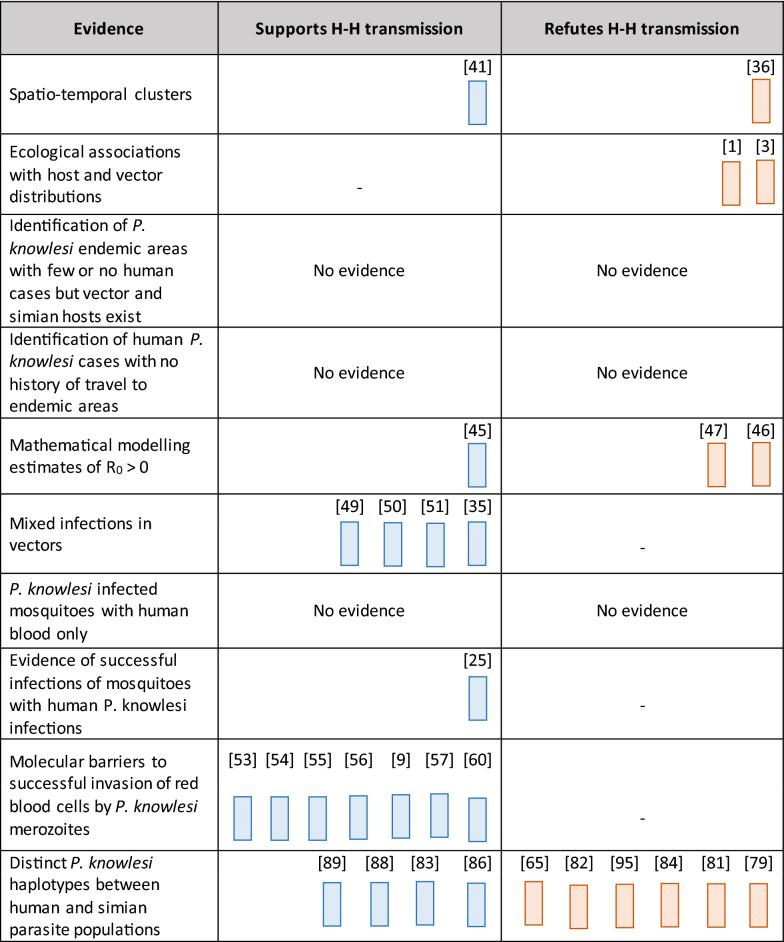
Harvest plot indicating all papers that support or refute sustained human-mosquito-human transmission within each evidence category, with the corresponding reference numbers

Conversely, 11 studies were identified which refuted the occurrence of sustained non-zoonotic transmission. One described clusters which did not support human-mosquito-human transmission, two indicated strong ecological associations between presence of simian hosts and human cases, two provided evidence from mathematical models refuting non-zoonotic transmission, and six described possible mixed haplotypes.

Overall, the evidence indicated that sustained non-zoonotic transmission is possible, but evidence of widespread occurrence is sparse. The evidence will be described in further detail below.

#### Evidence that *P. knowlesi* infections in humans can successfully infect malaria vectors

Following the report of the first naturally infected human case of *P. knowlesi* [24], research tried to identify the possibility of human-mosquito transmission of parasites. Soon after this, experimental evidence was reported showing successful infection of *Anopheles balabacensis* with parasites from a human case [25]. The study used human volunteers, which had been infected with *P. knowlesi*, to infect the vector. After a suitable incubation period had passed, these mosquitoes were allowed to feed on un-infected human volunteers. Mosquitoes were then dissected and blood samples from the human volunteers were taken to show that successful human infection had occurred via a vector. Early studies also identified the possibility of vector infections following human cases of other simian plasmodium species, notably *P. cynomolgi* and *Plasmodium inui* [26–28].

There has been limited research into gametocyte production, a key aspect in the parasite’s lifecycle related to non-zoonotic transmission, in *P. knowlesi* cases in humans. Research into infections in both natural and experimental simian hosts has identified 24-h cycles of gametocyte production, with peak densities occurring between midnight and 6 am [29, 30]. While this coincides with peak biting hours for some vector species, it contrasts with the reported peak human biting hours of the main vector species in Malaysian Borneo, *An. balabacensis*, which bites humans during crepuscular hours of 6 pm to 10 pm. The biting times observed in macaques, by contrast, have been shown to be late in the night; these peak biting times of the mosquitoes on macaques correspond to the peak times of gametocyte production observed in macaques [30–32]. Similarly, studies have also shown a relationship between increasing gametocyte densities in simian infections and infectiousness to mosquitoes [33]. However, equivalent research looking at human infections is lacking. Epidemiological and diagnostic studies have identified gametocytes from human cases during field studies [34, 35]. Although microscopic identification and diagnosis of gametocytes in human infections are challenging, molecular methods have been successful at identifying gametocyte presence. However, it is not known whether gametocytes identified using molecular methods are of sufficient density to be infectious to mosquitoes. Furthermore, no evidence was identified that described the peak production/density of gametocytes in human infections. As available data are from experimental human infections under laboratory conditions, it remains unknown how gametocyte densities vary in naturally infected humans over time and how gametocyte density relates to probability of transmission.

#### Spatiotemporal clusters of *P. knowlesi* cases consistent with human-mosquito-human transmission

Micro-epidemiological studies have helped identify small case clusters in various settings and have paid particular attention to potential transmission pathways. A study in north-eastern Sabah, in Malaysian Borneo, identified two family clusters. [5] In both cases, all family members presented with clinical symptoms on the same day, indicating that these cases were likely not part of the same non-zoonotic transmission chain. Members of the family affected in the first cluster reported no forest travel, but they mentioned macaque sightings around their dwelling. This, along with the age distribution, suggested that transmission took place in the peridomestic environment. Other studies have also identified vectors in peridomestic areas and infections in demographic groups not associated with occupational movements into forest or plantation areas [36–39]. Integrated analyses of vector and human mobility data indicated transmission most likely occurred at forest edges in close proximity to houses [40].

A second study, carried out in Sabang island in Aceh, Indonesia, also identified two small case clusters. [41] The first one was associated with a construction site where workers were staying overnight with no mosquito protection. This study reinforced previous knowledge about occupational forest transmission. Cases in the second cluster were all from the same family, who lived in a household on the forest fringes and reported regular macaque sightings around their home. The highlight of this cluster was the timings of the cases, which were indicative of transmission between family members as they all presented symptoms within a 12 day period. The final case presented with symptoms 12 days after the suspected index case and 3 days after the initial case had been diagnosed. This time-lag is potentially compatible with non-zoonotic transmission. However, evidence of nonzoonotic transmission would have been stronger if entomological and primatological investigations had also been conducted at the site to identify whether transmission was likely to have occurred within the area without a reintroduction from a zoonotic reservoir.

Although neither of these studies used quantitative methods or genomic analyses to assess possible transmission dynamics, they highlight the potential for non-zoonotic transmission. Further work applying quantitative methods to similar studies is needed, in particular models which incorporate spatio-temporal analysis of cases. These methods have been developed to reconstruct transmission chains of introductions or secondary cases following spillover [16, 42]. While these two papers provide evidence supporting (but not proving) human cases and speculation about potential non-zoonotic transmission, neither of these studies quantitatively assessed transmission. Existing unpublished case reporting data and surveillance records likely represent a rich data source to evaluate clusters consistent with chains of human-mosquito-human transmission.

#### Association of *P. knowlesi* cases with macaque and vector densities

The association of human *P. knowlesi* cases with wildlife host and vector densities increases the evidence for, but does not prove, transmission primarily driven by zoonotic spillover. At broad geographical scales, the distribution of human *P. knowlesi* cases correspond to the distributions of long-tailed and pig-tailed macaques and *Anopheles* Leucosphyrus vectors [1, 3]. Associations with habitats of other potential simian hosts and recently identified mosquito vectors have not been assessed.

Other studies have identified habitats associated with human *P. knowlesi* risks as indicators of potential contact with vector and wildlife species. Epidemiological studies have identified risk factors associated with both human exposure to and infection with *P. knowlesi* [7, 39, 43]. These papers highlight that men, particularly those of working age, have repeatedly been found to be at higher risk. Forest activities and contact with macaques are also both associated with exposure to *P. knowlesi*. Similarly, working in palm oil plantations has been identified as a risk factor for clinical cases. The peridomestic environment has also been shown to be important in determining where infections and exposures take place, with proximity to intact forest and rice paddies both increasing *P. knowlesi* risk. Although these factors do not directly support or refute non-zoonotic transmission, they are important for understanding where transmission is happening and who is affected.

#### Areas with vector and simian host cases but no human cases

No studies were identified which showed *P. knowlesi* in mosquitoes or non-human primates outside areas where human cases of *P. knowlesi* were reported (Additional file 3: Appendix 3, Additional file 4: Appendix 4). This likely reflects research and surveillance biases and cannot be used to conclusively prove or disprove sylvatic cycles of *P. knowlesi* transmission.

#### Human cases from non-*P. knowlesi* endemic areas

The identification of a human case of *P. knowlesi* without history of travel to currently known endemic regions, which are also enzootic, would be definitive proof of non-zoonotic transmission. While there is no systematic surveillance for *P. knowlesi* outside Southeast Asia, isolated cases have been detected in travellers returning from Asia. These isolated cases in travellers suggested that *P. knowlesi* infections are being captured by some surveillance systems, although recognizing *P. knowlesi* as a differential diagnosis and confirming with appropriate diagnostic methods outside of endemic areas is a substantial challenge [44]. Although many of these cases have had delayed diagnoses, their travel history suggested that they were infected in currently known endemic areas, and no onward cases have been reported in confirmed cases. This is particularly relevant as many of these areas where cases were identified lack the presence of suitable wildlife hosts and documented local transmission would have been proof of nonzoonotic transmission. Furthermore, no exported cases were identified in regions where malaria is already endemic, such as Africa or South America, despite substantial travel between Southeast Asia and these regions. This may be due to misdiagnosis as other malaria species are endemic within these regions rather than the lack of exported *P. knowlesi* cases..

#### Transmission modelling estimates of R_0_ > 0

Non-zoonotic transmission can be described by the reproductive number (R_0_), the expected number of secondary cases generated by one human case. Within this context, R_0_ > 0 indicates non-zoonotic transmission. 0 < R_0_ < 1 indicates weak transmission between humans, and R_0_ > 1 indicates sustained non-zoonotic transmission. Various modelling studies provide insight into the transmission dynamics of *P. knowlesi* by assessing reproductive rates under different scenarios. Theoretical mathematical modelling studies provide contextual evidence on whether human-mosquito-human transmission is consistent with observed parameter values, but the strongest evidence of non-zoonotic transmission would be estimates of R_0_ from empirical surveillance data. The earliest transmission model extended the standard Ross-MacDonald model usually used for malaria transmission by incorporating a specific compartment for simian host transmission [45]. Multi-host dynamics were modelled, comparing scenarios where humans were either non-competent (dead-end) or competent hosts. If humans are dead-end hosts, vectorial capacity has a non-linear relationship with mosquito preferences for biting humans; human cases cannot occur if vectors solely bite humans or solely bite macaques. Alternatively, if humans are competent hosts, vectorial capacity would increase with mosquito preferences for human hosts, with vectors exclusively biting human hosts most likely to maintain transmission of malaria in people. In order for a generalist mosquito vector species (i.e. a species biting both humans and macaques) to drive transmission, parasite transmission from macaques to humans must be twice as efficient as transmission between humans by a fully anthropophilic vector. However, if human population densities exceed those of macaque populations, transmission by a generalist vector will increase. This analysis highlights that although strong anthropophily is usually considered critical for a vector to effectively transmit malaria in people, generalist mosquito species may be more effective vectors of *P. knowlesi* in humans under certain conditions. This study identified key gaps in existing research (e.g. vector host preferences and exclusivity) and highlighted the need to explore human host competency.

A subsequent modelling study used a multi-host, multi-site transmission model to evaluate transmission in three potential land types: villages, farms and forests [46]. While this analysis was based on simulated data, only one of 1,046 plausible parameter sets evaluated was consistent with sustained non-zoonotic transmission and had a reproductive number in humans of 1.04. This single scenario was largely driven by very high vector-to-human and human-to-vector transmission probabilities. Estimates of the reproductive number in humans from all plausible scenarios ranged from 1.0 × 10^–5^ to 1.04, suggesting human-mosquito-human transmission is likely to occur only extremely rarely but is not impossible. Models were most sensitive to spatial overlap between humans and macaques, particularly macaque movements between farm and forest areas. A later study attempted to identify scenarios in which non-zoonotic transmission could occur by building on and refining these previous models. By maintaining certain parameters fixed and allowing other important variables to vary across a set scale, it estimated the average number of secondary human cases caused by a single macaque host case as well as those caused by a single human case. Using these terms and the range of values for the parameters included, the study concluded that, whilst plausible, sustained human-to-human transmission would rarely be taking place. It also showed that human infections were not playing a major role in parasite maintenance [47].

This study also highlighted the need for models which incorporate spatial heterogeneity of risk to improve understanding of the specific transmission dynamics. Indeed, previous spatial models have been used to create high resolution risk maps across the entirety of Southeast Asia by using both presence and absence of human cases. These have been helpful to identify which areas to prioritize for surveillance and control efforts [46]. However, a major caveat of these spatial models, which was recognized by the authors and explicitly stated in the study, was the assumption that no non-zoonotic transmission was taking place and *P. knowlesi* risks were solely determined by wildlife host and vector habitats. Mathematical transmission models incorporating spatial heterogeneities and allowing for both zoonotic and non-zoonotic transmission are needed to better understand where cases could occur. While the modelling frameworks developed could be updated with more recent empirical data, substantial gaps still remain in understanding vector biting preferences, simian host prevalence and contacts between vectors and hosts.

#### Presence of mixed infections of *P. knowlesi* with human malaria species in vector species and humans

Mixed infections of *P. knowlesi*, or indeed other simian malaria species, with human malaria species in mosquito vectors contributes to the theory that non-zoonotic transmission is taking place. The suggested mechanism is that mosquitoes are more likely to become infected with multiple *Plasmodium* species after biting an infected human host with a mixed malaria infection than to independently bite both infected simian reservoirs and humans simultaneously. The lack of identification of human malaria species circulating in macaques supports the theory that mosquitoes would not be infected with these *Plasmodium* species by biting non-human primates. Another plausible mechanism is quick, successive bites between human and simian hosts. It has been shown that these vectors have a probability of daily survival of between 0.83 and 0.87 depending on the region, supporting this possibility [48]. Similarly, mixed human infections could occur from bites from co-infected mosquitoes or separate bites from mosquitoes infected with simian and human species. A critical limitation to this source of evidence is that there are no indigenous human malaria cases currently reported in Malaysia, the epicentre of *P. knowlesi* transmission. Due to this, the lack of mixed infections in Malaysia since the last reports of human malaria transmission cannot be used as conclusive proof of zoonotic transmission.

Of particular importance are the findings of Nakazawa et al., Marchand et al. and Maeno et al. [49–51]. These studies have shown that *Anopheles dirus* mosquitoes in the Khanh Hoa province in South Vietnam have been found with mixed *Plasmodium* infection, with *P. knowlesi* found alongside *Plasmodium falciparum* and *Plasmodium vivax* (Table 3). Combinations have also included mixed infections of *P. knowlesi* and *P. vivax* with other potential zoonotic *Plasmodium* species, namely *P. cynomolgi* and *P. inui*. Although these studies were not able to determine the origin of bloodmeals from infected mosquitoes, these findings represent the importance of zoonotic transmission in this area. They also show the increasing risk of spillover from other simian *Plasmodium* species into local human populations (Table 4).

**Table 3:** Mixed infections reported in vector species, with numbers tested and numbers positive

Infection	Tested	Positive	Refs.
Mixed human—zoonotic			
* Pk* + *Pf*	72	1	[50]
* Pk* + *Pv*	6134	21	[50, 51]
* Pk* + *Pf* + *Pv*	80	8	[49, 50]
* Pk* + *Pv* + *Pcyn*	6062	2	[51]
* Pk* + *Pv* + *Pin*	6062	1	[51]
Other simian			
* Pk*	10 933	397	[31, 32, 36, 48, 50, 51, 99–104]
* Pk* + *Pcyn*	2937	4	[48, 102]
* Pk* + *Pin*	8969	5	[48, 51, 105]
* Pk* + *Pcyn* + *Pin*	1482	4	[48]
* Pk* + *Pct* + *Pin*	1482	1	[51]
* Pk* + *Pct* + *Pcyn* + *Pin*	1482	1	[48]

*Pk P. knowlesi,Pf P. falciparum, Pv P. vivax, Pcyn P. cynomolgi,Pin P. inui *and *Pct P. coatneyi* are accounted for in these mixed infections

**Table 4:** Mixed infections reported in humans

Infection	Number	Diagnosis	Year	Refs.
India				
* Pk* + *Pf*	1	PCR	2018	[106]
* Pk* + *Pf* + *Pv*	6	PCR	2018	[106, 107]
* Pk* + *Pv*	6	PCR	2018	[106, 107]
Indonesia				
* Pk* + *other*	97	PCR	2015	[108]
* Pk* + *Pv*	65	PCR	2015	[108]
Malaysia				
* Pk* + *Pf*	5	PCR	2004	[4]
* Pk* + *Pf* + *Pv*	1	PCR	2004	[4]
* Pk* + *Pv*	8	PCR	2004	[4]
* Pk* + *Pf*	6	PCR	2008	[103, 109]
* Pk* + *Pm*	4	PCR	2008	[103, 109]
* Pk* + *Po*	1	PCR	2008	[109]
* Pk* + *Pv*	7	PCR	2008	[103, 109]
* Pk* + *Pf*	3	PCR	2009	[110, 111]
* Pk* + *Pm*	3	PCR	2009	[110, 111]
* Pk* + *Pv*	86	PCR	2009	[110, 111]
* Pk* + *Pf*	1	PCR	2010	[112]
* Pk* + *Pv*	1	PCR	2010	[112]
* Pk* + *Pf*	9	PCR	2011	[5, 113]
* Pk* + *Pv*	36	PCR	2011	[5]
* Pk* + *Pf* + *Pv*	2	PCR	2011	[5]
* Pk* + *Pv* + *Pm*	2	PCR	2011	[5]
* Pk* + *Pm*	1	PCR	2011	[5]
* Pk* + *Pf*	1	Sequencing	2013	[114]
* Pk* + *Pv*	10	Sequencing	2013	[114]
* Pk* + *Pv*	3	NM-PCR	2014	[115]
* Pk* + *Pf*	6	PCR	2016	[116]
* Pk* + *Pf* + *Pv*	1	PCR	2016	[116]
* Pk* + *Pv*	6	PCR	2016	[116]
* Pk* + *Pcyn*	6	Sequencing	2017	[117]
* Pk* + *Pf*	12	Sequencing	2017	[117]
* Pk* + *Pv*	12	Sequencing	2017	[117]
Myanmar				
* Pk* + *Pf*	13	Sequencing	2008	[118]
* Pk* + *Pf* + *Pv*	2	Sequencing	2008	[118]
* Pk* + *Pv*	13	Sequencing	2008	[118]
* Pk* + *Pv*	1	Sequencing	2013	[119]
Thailand				
* Pk* + *Pv*	1	PCR	1996	[120]
* Pk* + *Pf*	5	PCR	2007	[121]
* Pk* + *Pv*	4	PCR	2007	[121]
* Pk* + *Pf*	6	PCR	2019	[120]
* Pk* + *Pf* + *Pv*	5	PCR	2019	[120]
* Pk* + *Pv*	4	PCR	2019	[120]
Vietnam				
* Pk* + *Pf*	1	PCR	2010	[50]
* Pk* + *Pf* + *Pv*	19	PCR	2010	[50]
* Pk* + *Pv*	12	PCR	2010	[50]

*Pk P. knowlesi, Pf P. falciparum, Pv P. vivax, Po P. ovale, Pm P. malariae *and *Pcyn P. cynomolgi *are accounted for in mixed infections

#### Evidence of human blood in mosquito vectors infected with *P. knowlesi*

Despite extensive review of entomological studies in the literature, no blood-fed mosquitoes infected with *P. knowlesi* were identified. Within Sabah, Malaysia, extensive studies were performed to identify resting locations of *P. knowlesi* vectors using different methods, but they failed to catch blood-fed *Anopheles* mosquitoes [52]. The lack of evidence of *P. knowlesi* in blood-fed mosquitoes neither proves nor disproves non-zoonotic transmission. However, it highlights the need for further entomological studies to identify the resting habitats of blood-fed *P. knowlesi* vectors.

#### Evidence of molecular barriers to successful invasion of red blood cells by *P. knowlesi*

For sustained non-zoonotic transmission to be possible, *P. knowlesi* must be able to invade human liver and red blood cells to complete the parasite life cycle. Early experimental studies into *P. knowlesi* focused on direct inoculation of parasites between hosts without assessing which mosquitoes were potential vectors in the wild. While limiting, these experimental studies did show that human-to-human transmission through direct blood inoculation is possible. Once evidence of natural infections taking place was uncovered, more emphasis was placed on determining the transmission dynamics of this malarial parasite [4, 24, 25]. Due to the relative feasibility of keeping *P. knowlesi* parasites in laboratory conditions, they were often used as models to understand how other *Plasmodium* parasites, in particular *P. vivax*, invaded human erythrocytes. This led to extensive experimental work carried out on this subject, which helped to identify specific proteins found on the cell surface that aid invasion by *P. knowlesi* [53–56].

One such experimental study has identified that, when kept in continuous culture, *P. knowlesi* parasites are able to adapt to invade and multiply in exclusively human red blood cell cultures [57]. This adaptation did not affect the parasite’s ability to invade red blood cells of macaque origin, with results indicating that invasion efficacy could be improved for both and maintained higher invasion efficacy for *M. fascicularis* red blood cells. This study also found that whilst *P. knowlesi* invasion of human red blood cells depends on the presence of the Duffy antigen, as shown extensively in other studies [58, 59], there is no difference in invasion and parasite growth between the three Duffy positive phenotypes. Further work on this human-adapted strain identified the need for a specific protein, normocyte binding protein Xa (NBPXa), for successful and efficient invasion of human red blood cells [60]. The adaptation to human red blood cells resulted from improved invasion of red blood cells, rather than improved intracellular growth. Deletion of this protein resulted in significantly reduced invasion capabilities in human red blood cell cultures, but there was no impact on *M. fascicularis* cultures. Experimental research has shown that *P. knowlesi* parasites under laboratory conditions have a preference for invading young erythrocytes over older ones, although this does not pose a significant barrier in invading older erythrocytes [61].

An important caveat to these results is the experimental nature of the findings. It is known that parasites maintained in continuous culture are primarily asexual blood-stage forms and can readily lose their ability to form sexual stage gametocytes, essential for mosquito transmission [57]. This means that natural infection dynamics might differ, but it should not distract from the fact that adaptation to sustained human transmission is possible. Nevertheless, it is also worth noting the higher invasion efficiency towards *M. fascicularis* red blood cells in both human-adapted and *M. fascicularis*-adapted strains, which may represent a bottle-neck for sustained human transmission. Although strains can become human-adapted, they will still be better at infecting *M. fascicularis* red blood cells than human blood cells, which will provide more possibility of transmission between the sylvatic host than between humans.

#### Genetic evidence of distinct parasite haplotypes in human and simian infections

Genetic studies are likely to provide one of the strongest sources of evidence to prove or disprove sustained non-zoonotic transmission. While many studies have focused on the genetics of *P. knowlesi*, a significant amount of these have centred around clinical questions. Substantial research has assessed the genetic variability of specific genes to identify suitable targets for vaccine development. Similarly, a large body of research has focused on identifying novel therapeutic targets in the parasite’s genome. Some of these studies have discussed their results in the context of transmission dynamics, but most have not explored this area in detail.

Studies have tried to estimate when the evolutionary divergence between *P. vivax* and *P. knowlesi* occurred, but varying estimates have been produced [62–64]. There is a need for further clarification on this point because this value is often used as a baseline to estimate other plasmodium evolutionary divergences.

Genetic studies have assessed the diversity of *P. knowlesi* genes by analysing the polymorphism within the gene and comparing sequences to other *Plasmodium* species (most frequently *P. vivax*) to determine the evolutionary pressure within this species. A small number of studies have identified possible balancing selection occurring at specific genes [65–67]. One study identified the possibility of balancing selection occurring at the apical membrane antigen 1 gene of *P. knowlesi* by comparing it to its orthologs in *P. falciparum* and *P. vivax*. The study was unable to conclude whether balancing selection was occurring within highly variable regions [68]. However, the majority of studies in which gene diversity was analysed concluded that purifying selection is currently taking place [69–81]. Possible reasons for this include immune evasion of both human and primate hosts, adaptation of genes to invasion of specific species and significant haplotype sharing between humans and macaques.

Studies researching the diversity within specific *P. knowlesi* genes have also helped identify separate clusters within the parasite population. Three broad groups have been identified by population genetic analysis: cluster 1, associated with long-tailed macaques (*M. fascicularis*) in Malaysian Borneo; cluster 2, associated with pig-tailed macaques (*M. nemestrina*) in Malaysian Borneo; and cluster 3, which is associated with both human and macaque cases in Peninsular Malaysia [65, 82–84]. The first two clusters are thought to have separated due to sympatric divergence between the two species of macaques and the ecological areas they inhabit, with pig-tailed macaques living mostly in remote forest areas and long-tailed macaques being found in both forested and urban areas, whilst the third is thought to have occurred due to allopatric separation as a result of the ocean barrier between Peninsular Malaysia and Malaysian Borneo. Although the separation of the clusters is clear, there has been some evidence of mixing between clusters 1 and 2, which is thought to have been caused by deforestation and the associated changes in vector populations [85]. Furthermore, parasites from cluster 1 have been more commonly found in human infections compared to those from cluster 2 [86]. Genetic analyses of human cases also identified some potential hybridization between the two main macaque clusters [87]. Although these results were still uncertain, they could indicate potential non-zoonotic transmission between humans.

These studies analysing the clustering found within the parasite population have also shed some light on the transmission dynamics, as is highlighted by Wilcox et al. [88]. They defined three possible phylogenetic trees, according to three separate possible transmission dynamics. If *P. knowlesi* were a strict zoonosis, isolated human strains would be found within macaque clades. Under the hypothesis of a host-specific disease, infections would only transmit within hosts which would create separate host-specific clades clustered together. If the disease were host specialized, host-specific strains would cluster separately, but some isolated human strains would cluster within macaque clades, representing limited zoonotic transmission. Using the context of these three hypotheses, these studies have found phylogenetic structuring of *P. knowlesi* populations between humans and macaques that could potentially contribute to evidence of human-to-human transmission. Although limited samples geographically restricted to Sarawak in Malaysian Borneo were used and these studies could not confirm whether these population differences were due to separate transmission pathways, this finding is potentially indicative of non-zoonotic transmission and should be a target for future research [88, 89].

Genetic analysis has also helped determine if there is any presence of drug resistance developing in *P. knowlesi* populations. This question is important for therapeutic reasons, but it also could help determine how much non-zoonotic transmission might be taking place, because drug resistance would only be expected to be selected for in human infections. Current evidence shows that no drug resistance is developing to the standard anti-malarial therapies in parasite populations [90]. Furthermore, mutations granting drug resistance to *P. falciparum* populations have not been found in *P. knowlesi* populations, and mixed infections with *P. knowlesi* and either *P. falciparum* or *P. vivax* do not seem to induce any drug resistance [91–93]. It is important to highlight that, whilst finding resistant mutations in *P. knowlesi* populations would suggest human-mosquito-human transmission is occurring, the lack of evidence of drug resistance cannot be used to prove an absence of non-zoonotic transmission. Further work is needed to aid surveillance of drug resistance mutations.

## Discussion

This systematic review has assessed the evidence available to determine if sustained human-mosquito-human transmission of *P. knowlesi* is taking place (Table 5). A novel framework was developed to integrate different evidence types, creating a template for studies evaluating transmission patterns of emerging diseases. Overall, evidence indicated that sustained non-zoonotic transmission is possible, but evidence of widespread occurrence is sparse. Although human-mosquito-human *P. knowlesi* transmission has been demonstrated experimentally, there is limited empirical or modelling evidence to suggest non-zoonotic transmission occurs naturally. Furthermore, while case reports are abundant and well documented, specific quantitative analyses to determine transmission routes have not been performed. These transmission pathways have major policy implications for international and national organizations, directly impacting design of anti-malarial control measures and malaria elimination certification in *P. knowlesi* endemic areas [9].

**Table 5:** Main conclusions from identified literature with accompanying qualitative assessment of evidence

Conclusion	Evidence
Experimental human-mosquito-human transmission has been demonstrated in laboratory settings	High consistency, limited evidence
Spatio-temporal clusters of human cases have been found which may be consistent with human-mosquito-human transmission but no quantitative analyses have been performed to confirm this	Medium consistency, limited evidence
Distribution of known natural hosts and vectors for *P. knowlesi* correlates with areas where human cases have been reported	High consistency, robust evidence
No secondary *P. knowlesi* cases have been reported outside endemic areas without a history of travel	Medium consistency, limited evidence
The lack of *P. knowlesi* cases in malaria endemic areas is most likely due to detection bias and misdiagnosis	High consistency, limited evidence
Models suggest human-mosquito-human transmission is unlikely but still plausible within observed parameters	High consistency, medium evidence
Models suggest reproductive rates are highly sensitive to contact patterns between simian hosts, vectors and people as well as vector biting preferences and likely to be highly affected by land use change	High consistency, robust evidence
Mixed infections with *P. knowlesi* and human malaria species have been reported in both humans and known natural vectors across various countries in South-East Asia	High consistency, robust evidence
*P. knowlesi* parasites can adapt to exclusive human red blood cell culture, invading and multiplying successfully for multiple generations	High consistency, medium evidence
*P. knowlesi* parasites have a preference for invading young human erythrocytes, although this does not pose a significant barrier to invasion	Medium consistency, medium evidence
Multiple invasion pathways have been identified, with a range of specific proteins aiding cell invasion. This shows there are no molecular barriers to invasion of human erythrocytes other than the requirement of Duffy antigens	High consistency, robust evidence
There are genetically distinct subpopulations of *P. knowlesi* parasites associated with different macaque populations and human cases from different geographical areas but no clear evidence of host specific circulation as would be expected with widespread non-zoonotic transmission	Low consistency, medium evidence
There is no evidence of drug resistance in *P. knowlesi*	High consistency, robust evidence

The strongest evidence of the feasibility of non-zoonotic *P. knowlesi* transmission is from experimental human studies; these demonstrated the parasite can replicate in human hosts and can result in subsequent human infections through mosquitoes [25]. Additionally, laboratory studies have shown that cultured parasites can adapt to reproduce asexually within human red blood cell cultures without needing macaque red blood cells [57]. Furthermore, these studies have not been able to identify any significant barriers to human red blood cell invasion, with a range of proteins facilitating multiple invasion pathways [53–57, 60, 94].

By contrast, genetic and mathematical modelling studies present a more nuanced picture. Genetic studies have identified genetically distinct *P. knowlesi* subpopulations associated with different macaque species and geographical regions, with limited but unclear evidence of host-specific circulation, which would be expected if non-zoonotic transmission were occurring [65, 79, 81–84, 87–89, 95]. A lack of evidence for drug resistance in *P. knowlesi* populations suggests that transmission between humans is unlikely [90], although drug resistance can take some time to develop. Similarly, mathematical models of *P. knowlesi* transmission have found that human-mosquito-human transmission is within the range of plausible parameters but highly unlikely [46, 47]. The lack of any human *P. knowlesi* infections reported outside areas with macaques supports transmission primarily occurring through spillover, although this is subject to major limitations in testing and reporting.

A main output of this review is the identification of key knowledge gaps. Mathematical modelling studies have highlighted key parameters that are still unknown, such as reservoir infection prevalence, vector biting preferences and contact patterns between reservoirs and human hosts. Substantial questions remain about vector species, bionomics and biting behaviour. While *P. knowlesi* infections have been identified in *Anopheles* species outside the Leucosphyrus Group, these species have not been identified as vectors through confirmation of *P. knowlesi* sporozoites and oocysts present in the mosquito species [96]. Additionally, the vector dynamics driving sylvatic transmission between wildlife reservoirs remain largely unknown. Similarly, despite relatively strong experimental evidence of non-zoonotic transmission, there are limited studies describing gametocyte dynamics in human infections. Further research is necessary to identify if all human infections have gametocytes and the timing and density of gametocyte production. The gametocyte density threshold needed for mosquitoes to become infected is likely to be similar to that of other malarias, requiring a minimum of 1 male and 1 female gametocyte in a 2–3 ul mosquito blood meal. Therefore, higher densities of gametocytes are likely to be associated with increased chance of transmission success.

The complexity of the evidence assessed highlights the challenges of characterizing transmission of emerging diseases. While contact tracing and detailed follow ups of human cases can be used to reconstruct transmission chains of directly transmitted diseases, these methods are more challenging for vector-borne diseases where transmission is not directly observed. Identifying transmission chains can be made more difficult by frequent misdiagnoses or imperfect surveillance data aggregated at administrative levels [11]. Methodological developments in mathematical modelling approaches provide opportunities to estimate spillover and non-zoonotic reproductive rates from imperfectly detected surveillance data of vector-borne diseases [42, 97, 98]. These approaches have not yet been applied to *P. knowlesi* cases but would yield substantial insights into disease dynamics. Within this systematic review, a methodological framework has been developed to evaluate transmission dynamics of vector-borne zoonotic diseases and collect data on key parameters which could inform future modelling attempts.

While this review is the first to systematically evaluate *P. knowlesi* transmission pathways, it has several important limitations. Notably, the reviewers did not have access to unpublished data such as routinely collected surveillance data on malaria cases or unpublished reports of macaques or mosquito studies. Although abstracts were screened in multiple languages, it was not feasible to include all languages from *P. knowlesi* endemic areas, and some case reports or studies may have been excluded. Furthermore, the heterogeneity of evidence types precluded the use of standard approaches for quantitatively assessing evidence (e.g. GRADE). Instead, evidence assessment methods were adapted from those used in climate science, a field with similar difficulties in combining disparate evidence sources with varying levels of certainty [23]. This review provides a template for similar studies evaluating multiple evidence categories.

Despite these limitations, the evidence synthesis demonstrates that sustained human-mosquito-human transmission chains are indeed possible, but currently available evidence indicates that, if these transmission events are occurring, they might be rare. This review identifies specific areas that need further research, in particular quantitative analyses to disentangle transmission dynamics by combining epidemiological and entomological surveillance, and ecological studies to develop a better understanding of the sylvatic cycle. Determining the exact transmission dynamics of this parasite is of great importance to policy development and could have significant implications for both global and local malaria elimination efforts.

## Supplementary Information

**Additional file 1**. Systematic review protocol.

**Additional file 2**. Evidence synthesis.

**Additional file 3**. Human cases of *P. knowlesi*

**Additional file 4**. Host and vector infections with *Plasmodium* species.

## Data Availability

All data generated or analysed during this study are included in this published article and its Additional files.

## Abbreviations

CoCoPop: Condition, context, population
ERG: Expert Review Group
IPCC: Intergovernmental Panel on Climate Change
NBPXa: Normocyte binding protein Xa
PRESS: Peer Review of Electronic Search Strategies
PCR: Polymerase chain reaction
R_0_: Reproductive number
SWiM: Synthesis without meta-analysis
WHO: World Health Organization
