# Supplementary material for: Is there evidence of sustained human-mosquito-human transmission of the zoonotic malaria Plasmodium knowlesi? A systematic literature review
Source: Malar J. 2022 Mar 17;21:89. doi: 10.1186/s12936-022-04110-z (PMC8929260; doi:10.1186/s12936-022-04110-z)
Supplement: Supplementary file 1 — Additional file 1. Systematic review protocol. [file 12936_2022_4110_MOESM1_ESM.docx]

**What evidence is there of sustained human-mosquito-human transmission of *Plasmodium knowlesi*? A systematic review protocol**

*Authors: Pablo Ruiz Cuenca, Kimberly Fornace, Chris Drakeley*

*Support: Funds provided by WHO*

# Introduction

**Rationale**

Zoonotic malaria, caused by the parasite *Plasmodium knowlesi*, has increasingly become a public health concern in South East Asia. It is maintained in the wild by non-human primates (including macaques) and is transmitted by mosquitoes from the Leucosphyrus group of *Anopheles*. The geographic distribution of the disease is limited to areas where both primate hosts and vectors are present, mostly affecting individuals who live in or travel through forests and forest fringes. ^1^ The incidence has been increasing in Malaysia, with cases caused by *P. knowlesi* being the predominant cause of indigenous malaria in the country since 2017. ^2^

As an emerging infectious disease, zoonotic malaria may be classified into 5 stages based on its epidemiological dynamics (Figure 1). ^3^ Stages 1 and 2 represent pathogens which are found in animals and have either not been found to naturally infect humans or have not been found to cause secondary human infections. Stage 3 pathogens are those that can cause secondary human infections but can only undergo a limited number of cycles. Stage 4 represents those pathogens that have a natural cycle of infecting humans from the primary animal host and can produce long sequences of secondary human cases. These are divided further into subgroups according to the importance of the animal and human cycles. Stage 5 pathogens are those that originated in animals but are now exclusively found in humans.

Stages 1 and 2 are not considered human pathogens, whilst stages 4 and 5 do fall into this category with important implications for control measures and, ultimately, elimination and eradication. The implications of pathogens which are classified within stage 3 are less clear, requiring further clarifications to inform control policies.

***Figure 1*** *– Depiction of suggested stages of an animal pathogen evolving to cause disease in humans. From Wolfe et al, Nature 2007.* ^3^

In 2017, a WHO evidence review group (ERG) examined the available evidence on zoonotic malaria to determine which stage it could be classified as. It concluded that, given there was limited evidence of sustained human transmission following an initial spillover, it should be considered primarily a zoonotic infection and classified within stage 2. However, if this were to change to stage 3 or above, criteria for malaria elimination certification would have to be revisited. The ERG also concluded that further research was necessary, as significant knowledge gaps were identified, and categorised the necessary evidence that would prove or refute sustained human transmission.

**Objectives**

The purpose of this systematic review is to examine current evidence to determine if any human-mosquito-human transmission of *Plasmodium knowlesi* occurs and if sustained transmission, defined as multiple generations of human cases with no spillover from macaques, is possible. This will inform WHO’s decision to classify zoonotic malaria and help determine appropriate elimination and eradication strategies and targets.

Furthermore, to help inform transmission models distinguishing zoonotic from non-zoonotic transmission, we will assemble data on the distribution and abundance of specific simian host and vector species. However, as this is not the main focus of the review, this will become the secondary objective.

# Methods

We will adapt the CoCoPop framework (Condition, Context, Population) suggested for prevalence and incidence questions to create our question and guide our work. ^4^ Given the broad scope of the question, the evidence required was categorised by the WHO evidence review group.

Evidence suggestive of sustained human transmission chains was defined as:

- Epidemiological evidence
  - Evidence of R_0_ >= 1in human population
  - Identification of space-time clusters consistent with human-mosquito-human transmission, including human case reports
- Laboratory evidence
  - Mixed (human-zoonotic) infections in mosquitoes
  - *P. knowlesi* infected mosquitoes with human blood only
  - Human infections successfully infecting malaria vectors (focus on Leucosphyrus group)
  - Demonstrating distinct haplotypes between parasites infecting humans and macaques
- Environmental/ecological evidence
  - Simian host distribution and abundance consistent with no possible spillover events
  - Vector distribution and density, specifically Leucosphyrus group, proving no possibility of spillover from simian hosts
- Other suggestive evidence, for example:
  - *Plasmodium cynomolgi* research
  - Other regional zoonotic malarias

Additionally, evidence that refuted the possibility of sustained human transmission included:

- Laboratory evidence
  - Identifying molecular barriers to successful invasion of human red blood cells
- Epidemiological evidence
  - Prevalence of ligands associated with molecular barriers to parasitic invasion of human red blood cells

**Eligibility criteria**

Types of studies

We will only exclude literature reviews from our search. Otherwise, there will be no exclusions on the types of studies reviewed. These will include, but are not limited to, epidemiological observational and interventional studies, laboratory studies and modelling studies. We will also perform a search of the grey literature for any suitable evidence, including human case reports and vector and simian host distributions.

Language

We will conduct the search in English but will be aiming to include studies published in any language. We are able to directly consider sources in English, Spanish, French and Malay. For those in languages we are not familiar with, support with screening and/or translating will be sought from our professional networks.

Condition

The search will focus on *P. knowlesi* research but will also include relevant work carried out with regional *Plasmodium* species which could be suggestive of sustained human transmission chains. We will limit diagnoses to molecular confirmation methods. We will consider genomic sequencing to be the strongest evidence of a positive diagnostic followed by PCR.

Context

In light of the epidemiology of zoonotic malaria, our interest is focused on understanding if there are biological reasons that support or hinder sustained human transmission, and if this is occurring naturally already. Given the complexity of studying this phenomenon, we will be using studies carried out in both controlled laboratory conditions and in the field, and will not be restricting based on location. We will also be looking for any suggestive evidence produced by modelling studies.

Population

No exclusion criteria will be applied to populations included in the review.

**Information sources**

The following databases will be used to search for published research:

- Medline
- EMBASE
- Web of Science

We will also include searches for grey literature, including reports and data sets on human and simian hosts and vectors from the following organisations:

- ProMed
- IUCN
- PREDICTS project
- Cambridge Conservation Initiative
- Zoological Society of London
- Zenodo

**Search strategy**

As there is limited published research on *P. knowlesi*, we will be using a wide search strategy in hopes of capturing all necessary evidence. The search strategy has been peer reviews by an Information Scientist from the London School of Hygiene and Tropical Medicine library, using PRESS peer review standard and guidelines.^5^

An example of the search strategy we will use in Medline is detailed below:

1     Plasmodium knowlesi/
2     plasmodium knowles*.mp.
3     1 or 2

4     Zoonoses/
5     catarrhini/ or cercopithecidae/ or cercopithecinae/ or exp macaca/
6     (monkey* or simian* or zoono* or macaca or macaque*).mp.
7     4 or 5 or 6

8     (malaria* or plasmodium).mp.
9     Malaria/
10     8 or 9

11     7 and 10
12     3 or 11

**Study records**

Selection process

All published literature will be imported into a reference manager, which will be used to de-duplicate any papers. Titles and abstracts will then be screened and removed as necessary. We will utilise the Rayyan tool for screening titles and abstracts. ^6^ One reviewer will screen all titles and abstracts, whilst the second reviewer will assess those that have been excluded. Once the final list of texts has been agreed, both reviewers will review all texts concurrently.


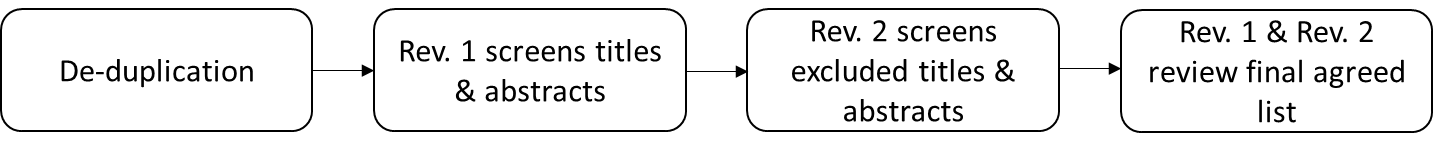


Data extraction and management

A standardised form will be used to extract necessary data from published literature. Given the varied evidence we will be collecting, the synthesis without meta-analysis (SWiM) framework will be used, applying vote counting to combine evidence. ^7^ Using the categories of evidence outlined above, the evidence suggestive of sustained human transmission will be considered a positive vote (▲) whilst the evidence refuting sustained transmission will be considered a negative vote (▼).

The data extraction form will include year of publication, population, setting (including georeferenced data if possible), diagnostic used (if applicable), category and direction of evidence (vote counting). A separate form will be used to extract necessary data from grey literature, including host and vector data to improve model parameters. Sample extraction forms are included in Appendix II.

**Outcomes and prioritization**

Our main outcomes will be guided by the evidence categories defined above. We will prioritise definitive evidence of sustained human transmission, but will also seek suggestive evidence, such as modelling work performed with host and vector data.

Guided by our secondary objectives of finding parameters to improve transmission models, our secondary outcomes will be identifying data sets and evidence to improve parameterisation of models. These parameters are detailed in Appendix I.

**Risk of bias in individual studies**

As we are expecting a broad range of types of studies, the risk of bias will be assessed according to each category of evidence. We will assess what types of evidence need to be assessed before applying specific tools to assess risk of bias. Examples are included in Table 1.

***Table 1*** *– Possible tools assessing risk of bias*

| Tool | Type of study |
| --- | --- |
| Cochrane RoB 2 | Experimental epidemiological study |
| Newcastle - Ottowa Scale (NOS) | Case-control studies |
| QUADAS-2 | Diagnostic studies |

**Data Synthesis**

Data will be synthesised using the SWiM framework, combining evidence by vote counting. This will be done narratively, graphically (eg harvest plots^8^) and in tabular form to help interpret results. Given the comprehensive nature of the evidence we will be searching, we will not be assessing meta-biases of the studies included.

**Confidence in cumulative evidence**

Given the range of evidence we will be searching, we will be using the Grading of Recommendations Assessment, Development, and Evaluation (GRADE) approach applicable to each type of study (epidemiological, diagnostic and modelling). ^9,10^

# References

1. Fornace, K. M. *et al.* Environmental risk factors and exposure to the zoonotic malaria parasite Plasmodium knowlesi across northern Sabah, Malaysia: a population-based cross-sectional survey. *Lancet Planet. Heal.* **3**, e179–e186 (2019).

2. William, T. *et al.* Changing epidemiology of malaria in Sabah, Malaysia: Increasing incidence of Plasmodium knowlesi. *Malar. J.* **13**, 390 (2014).

3. Wolfe, N. D., Dunavan, C. P. & Diamond, J. Origins of major human infectious diseases. *Nature* **447**, 279–283 (2007).

4. Munn, Z., Moola, S., Lisy, K., Riitano, D. & Tufanaru, C. Methodological guidance for systematic reviews of observational epidemiological studies reporting prevalence and cumulative incidence data. *Int. J. Evid. Based. Healthc.* **13**, 147–153 (2015).

5. McGowan, J. *et al.* PRESS Peer Review of Electronic Search Strategies: 2015 Guideline Statement. *J. Clin. Epidemiol.* **75**, 40–46 (2016).

6. Ouzzani, M., Hammady, H., Fedorowicz, Z. & Elmagarmid, A. Rayyan-a web and mobile app for systematic reviews. *Syst. Rev.* **5**, 210 (2016).

7. Campbell, M. *et al.* Synthesis without meta-analysis (SWiM) in systematic reviews: Reporting guideline. *BMJ* **368**, (2020).

8. Ogilvie, D. *et al.* The harvest plot: A method for synthesising evidence about the differential effects of interventions. *BMC Med. Res. Methodol.* **8**, 8 (2008).

9. Guyatt, G. H. *et al.* GRADE: An emerging consensus on rating quality of evidence and strength of recommendations. *BMJ* **336**, 924–926 (2008).

10. Schünemann, H. J. *et al.* Grading quality of evidence and strength of recommendations for diagnostic tests and strategies. *Chinese Journal of Evidence-Based Medicine* **9**, 503–508 (2009).

# Appendix I – Model Parameters

Data and evidence will be sought to help improve and inform the following model parameters:

- Human host-parasite interactions
  - Parasite virulence
  - Parasite binding to human red blood cells
  - Duration of infection/infectivity
- Human host-vector interactions
  - Human-mosquito infectiousness
  - Human host biting rate
  - Biting preference
  - Mosquito:humans ratio
- Vector-parasite interactions
  - Mosquito lifespan
  - Reproductive ability of parasite inside mosquito
  - Duration of development in mosquito
- Simian host-vector interactions
  - Simian ecology
  - Biting rate
  - Biting preference
  - Duration of infection/infectivity

# Appendix II – Sample extraction forms

Evidence synthesis:

| ID | Title | Abstract | Authors | Year | Journal | Evidence category (epi/lab/eco) | Sub-category | Risk of bias | Certainty of evidence | Direction of evidence (vote counting: 🡩, - , 🡫) |
| --- | --- | --- | --- | --- | --- | --- | --- | --- | --- | --- |
|  |  |  |  |  |  |  |  |  |  |  |
|  |  |  |  |  |  |  |  |  |  |  |
|  |  |  |  |  |  |  |  |  |  |  |
|  |  |  |  |  |  |  |  |  |  |  |

Vector/host data extraction:

| ID | Vector/Host | Species | Presence/abundace | Number | Date | Country | Location | Latitude | Longitude | Source_ID | Source |
| --- | --- | --- | --- | --- | --- | --- | --- | --- | --- | --- | --- |
|  |  |  |  |  |  |  |  |  |  |  |  |
|  |  |  |  |  |  |  |  |  |  |  |  |
|  |  |  |  |  |  |  |  |  |  |  |  |
|  |  |  |  |  |  |  |  |  |  |  |  |
